# Isolated Pancerebellar Syndrome With anti-GQ1b IgG Positivity: A Case Report

**DOI:** 10.1177/19418744251367176

**Published:** 2025-08-06

**Authors:** Iyas Daghlas, Liza Solovey, Vanja C. Douglas

**Affiliations:** 1 UCSF Weill Institute for Neurosciences, Department of Neurology, 8785 University of California San Francisco, San Francisco, CA, USA

**Keywords:** anti-GQ1b, case report, cerebellar ataxia, EBV, GBS, Miller-Fisher

## Abstract

**Background:**

Ataxia is a hallmark of the anti-GQ1b antibody syndrome, though it is unclear whether this symptom arises primarily from sensory nerve injury or from cerebellar involvement. We report a case of a patient with a clinically isolated post-infectious pancerebellar syndrome with anti-GQ1b antibody positivity.

**Case Presentation:**

A 22-year-old previously healthy woman presented with acute-onset, progressive imbalance, limb ataxia, and dysarthria following an upper respiratory tract infection. She had no paresthesias, numbness, or diplopia. Neurological examination revealed an isolated pancerebellar syndrome including ocular and limb dysmetria, intention tremor, and gait ataxia. All sensory modalities were unaffected, reflex testing was normal, and there was no ophthalmoplegia. In-hospital serum and CSF testing was unremarkable, and brain magnetic resonance imaging was normal. The patient was empirically treated with intravenous immunoglobulin. Her symptoms were moderately improved by discharge (hospital day 5), and fully resolved several months later. Serological testing sent during the hospitalization subsequently revealed anti-GQ1b IgG positivity.

**Conclusions:**

This case expands the spectrum of anti-GQ1b syndrome to include isolated cerebellar ataxia, suggesting that ataxia in this syndrome can have cerebellar origin. Clinicians should consider anti-GQ1b testing in cases of acute cerebellar ataxia following infection.

## Background

The syndrome of acute ophthalmoplegia, ataxia, and areflexia was first described by Miller Fisher in 1956.^1^ Subsequent investigations demonstrated that patients with this syndrome harbor serum IgG antibodies against the GQ1b ganglioside.^2^ The phenotypic spectrum of the anti-GQ1b syndrome has since broadened to include isolated elements of the clinical triad, as well as additional manifestations such as encephalopathy and optic neuropathy.^2^

At the time of Miller Fisher syndrome’s initial description, the associated ataxia was favored to be of cerebellar origin.^1^ However, the concomitant areflexia and sensory loss characteristic of this syndrome has fueled debate as to whether the ataxia may instead be of sensory origin.^1,3-5^ To our knowledge, there are no previously reported cases of anti-GQ1b syndrome presenting with isolated ataxia. Such a presentation would provide compelling evidence for a cerebellar etiology to the ataxia in the anti-GQ1b syndrome. In this report, we describe a case of postinfectious, isolated pancerebellar syndrome with anti-GQ1b IgG antibody positivity. We discuss the clinical implications for expanding the recognized spectrum of the anti-GQ1b syndrome, as well as the mechanistic insights this case offers into the pathophysiology of ataxia in this syndrome.

## Case Presentation

A 22-year-old woman presented to our emergency department with 5 days of new progressive clumsiness, gait instability, and slurring of her speech. She also reported nausea and a new headache. Her condition deteriorated, ultimately resulting in inability to walk and unintelligible speech, prompting her to seek emergency evaluation. Review of systems was notable for an upper respiratory tract infection that preceded onset of symptoms by 1 week; there was no associated diarrhea. The patient denied numbness, paresthesias, diplopia, or any preceding rheumatologic symptoms. She had no significant medical history, took no medications, and denied toxic ingestions or recreational drug use. She endorsed a family history of lupus cerebritis in her half-sister.

On initial physical examination, the patient had normal vital signs. Arousal, orientation, and language function were normal. Cranial nerve examination demonstrated bilateral ocular dysmetria, saccadic intrusions, and scanning speech. Pupils were of normal size and equally reactive, eye movements were full without nystagmus, and facial strength and sensation were normal. Power was full in the proximal and distal upper and lower extremities. Sensation was normal in all extremities to light touch, vibration, and pinprick. Joint position sense was preserved in the hands and feet. Deep tendon reflexes were symmetric and ranged from 1-2+ at the biceps, brachioradialis, triceps, patellae, and ankles. Cerebellar examination revealed intention tremor on bilateral finger-nose-finger testing, bilateral dysmetria and dysdiadochokinesia in the upper extremities, and impaired heel-shin coordination. The patient exhibited a cerebellar ataxic gait requiring walker assistance for safe ambulation.

Serum electrolytes, liver function testing, thyroid stimulating hormone, and blood counts were normal. Urine pregnancy and toxicology tests were negative. Further testing for infectious and autoimmune etiologies is summarized in Table 1 and was only notable for low-grade positivity of several inflammatory markers including antinuclear antibody (1:160), erythrocyte sedimentation rate (25 mm/h), and proteinase 3 antibody IgG (31 AU/mL); however, there were no supportive findings for lupus or vasculitis on review of systems, laboratory investigations (Table 1), or on systemic imaging (eg, no pulmonary or sinus abnormalities). CSF testing on the first day of hospitalization (day 6 of illness) revealed no abnormal findings (Table 2). Magnetic resonance imaging (MRI) of the brain performed with and without gadolinium contrast was normal, with no enhancement of the cerebellum or cranial nerves. CT of the chest, abdomen, and pelvis showed no evidence of underlying malignancy, lymphadenopathy, or hepatosplenomegaly.

**Table 1.** Results From In-Hospital Serologic Testing.

Timing of Result	Serum Test	Result (Normal Range)
In-hospital	HIV 1/2 antigen and antibodies	Negative
	*T. pallidum* antibodies	Negative
	Monospot test	Negative
	C-reactive protein	2.4 mg/L (0-10)
	Erythrocyte sedimentation rate	**25 mm/h (0-20)**
	Antinuclear antibody	**Speckled 1:160 (<1:80)**
	C3 complement	154 mg/dL (82-170)
	C4 complement	36 mg/dL (12-40)
	Angiotensin converting enzyme	19 U/L (16-85)
	SSA-60 IgG, SSB IgG, double-stranded DNA antibody, rheumatoid factor, beta-2-glycoprotein IgG, IgM, cardiolipin IgG, IgM, myeloperoxidase IgG, ANCA IFA	Below reference range (varied)
	Proteinase 3 antibody IgG	**31 AU/mL (<26)**
	Vitamin B12	**1075 pg/mL** (211-911)
	Comprehensive toxin screen	None detected
Post-discharge	GM1 IgG, GM1 IgM, GQ1b IgM, GD1b IgG, GD1b IgM	All <10 IV (0-50)
	GQ1b IgG antibody	**120 IV (0-50)** ^**3**^
	Mayo clinic autoimmune encephalitis panel^a^ (Test ID: ENS2)	All antibodies nonreactive
	EBV DNA	Negative
	EBV viral capsid antigen IgG	**658 U/mL (<23)**
	EB nuclear antigen IgG	**>600 U/mL (<23)**
	EBV viral capsid IgM	**48.3 U/mL (<45)**
	EBV early diffuse IgG	<5 U/mL (<12)

Abnormal laboratory values are bolded.

^a^The Mayo Clinic panel tests for antibodies against the following antigens: AMPA-R, Amphiphysin, AGNA-1, ANNA-1, ANNA-2, ANNA-3, CASPR2, CRMP-5, DPPX, GABA-B-R, GAD65, GFAP, mGluR1, IgLON5, LGI1, Neurochondrin, NIF, NMDA-R, PCA-Tr, PCA-1, PCA-2, PDE10A, Septin-7, and TRIM46. EBV: Epstein-Barr virus; HSV: herpes simplex virus; VZV: varicella zoster virus.

**Table 2.** Results From In-Hospital Cerebrospinal Fluid (CSF) Diagnostic Testing.

Timing of Result	CSF Test	Result (Normal Range)
In-hospital	Total nucleated cells^a^	<6^2^/μL (<6)
	Red blood cells	**3/μL (<1)**
	Glucose	50 mg/dL (40-70)
	CSF protein	30 mg/dL (14-45)
	Bacterial culture and gram stain	Negative
	HSV-1 and HSV-2 PCR	Negative
	VZV PCR	Negative
Post-discharge	VZV IgM	0 ISR (<0.9)
	VZV IgG	<10 IV (<134.9)
	IgG index	0.52 (0.28-0.66)
	Unique oligoclonal bands	0 bands (0-1)
	Mayo autoimmune encephalitis panel (Test ID: ENC2)^b^	All antibodies nonreactive

Abnormal laboratory values are bolded.

^a^Our reference laboratory does not report the nucleated cell count in the CSF if the number is below 6. HSV: herpes simplex virus; VZV: varicella zoster virus.

^b^The Mayo Clinic panel tests for antibodies against the following antigens: AMPA-R, Amphiphysin, AGNA-1, ANNA-1, ANNA-2, ANNA-3, CASPR2, CRMP-5, DPPX, GABA-B-R, GAD65, GFAP, mGluR1, IgLON5, LGI1, Neurochondrin, NIF, NMDA-R, PCA-Tr, PCA-1, PCA-2, PDE10A, Septin-7, and TRIM46.

Given the disabling nature of symptoms and a possible post-infectious immune-mediated mechanism, we empirically treated the patient with intravenous immunoglobulin (IVIG). This was dosed at 2 grams per kilogram administered over 3 days, along with intravenous thiamine (500 mg 3 times daily for 3 days), followed by oral thiamine (100 mg daily). All serum and CSF antibody testing was sent prior to IVIG treatment.

Throughout the hospitalization, the patient demonstrated progressive clinical improvement and engaged effectively with rehabilitation services. Examination on the day of discharge (hospital day 5, illness day 10) showed minimal ocular dysmetria and significant improvement in dysarthria and dysmetria. She was able to return home with a walker for ambulation assistance, with plans for outpatient physical therapy and follow-up in the neurology clinic.

At 1-month clinic follow-up, the patient was able to tandem gait and had only mild residual dysarthria and dysmetria. Review of laboratory results that were pending at discharge revealed negative results for serum and CSF autoimmune and paraneoplastic antibodies testing (Table 1). An Epstein-Barr virus (EBV) antibody panel showed findings consistent with recent vs remote infection (Table 1). The serum anti-GQ1b IgG antibody, performed via semi-quantitative enzyme-linked immunosorbent assay at ARUP laboratories, returned positive with an index value of 120 (reference range: index value 0-50). At follow-up several months later, the patient had fully returned to her baseline functional status, with no residual deficits or recurrence of symptoms.

## Discussion and Conclusions

To the best of our knowledge, this is the first documented case of anti-GQ1b IgG antibodies associated with isolated ataxia. There was no evidence of sensory dysfunction on history and physical examination. Moreover, several findings were specific to cerebellar dysfunction, including scanning speech and ocular dysmetria. We therefore propose that this case constitutes evidence supportive of a cerebellar origin of ataxia in some patients with serum anti-GQ1b antibodies. This finding aligns with prior work which has provided alternative sources of evidence for cerebellar involvement in patients with syndromes attributed to anti-GQ1b antibodies. Murine studies have shown evidence of anti-GQ1b antibodies binding to cerebellar cells,^6^ and anti-GQ1b antibodies have been shown to bind to the human cerebellar molecular layer.^7^ A small, longitudinal study of transcranial magnetic stimulation over the cerebellum in patients with Miller Fisher syndrome showed findings consistent with cerebellar dysfunction.^8^ Finally, studies using MR spectroscopy^5^ and (18)F-fluorodeoxyglucose-positron emission tomography^9^ have shown transient cerebellar changes in patients with anti-GQ1b syndromes.

We acknowledge methodological limitations, alternative interpretations, and atypical aspects of this case. First, as a single case report, our findings require validation through independent replication. Second, although we detected anti-GQ1b antibodies, we lack definitive evidence of their pathogenicity in this presentation. Nevertheless, it is notable that these antibodies are highly specific and have not been documented in healthy controls.^2^ Third, the patient lacked albuminocytologic dissociation, though normal protein is often seen in patients who display partial features of the Miller Fisher triad or who present early in the disease course.^2,10^ Fourth, electrophysiological data were not obtained for this patient, as in-hospital nerve conduction studies were not clinically indicated in the absence of sensory symptoms. Fifth, although we suspect that the patient’s recent infection may have been the immunological trigger for GQ1b antibody production,^11^ we cannot exclude the possibility that this case represented a post-infectious cerebellitis due to another pathogenic antibody, wherein anti-GQ1b positivity represented an epiphenomenon rather than the primary pathogenic mechanism. Similarly, we cannot exclude direct cerebellar infection by EBV as we did not perform EBV testing on the CSF samples. However, several features argue against this possibility, including the delayed timing of neurological symptoms in relation to infectious symptoms, the absence of CSF pleiocytosis and MRI abnormalities,^12^ and the positive EBV nuclear antigen (which is not produced in acute infection). Sixth, we are uncertain whether clinical improvement was attributable to IVIG therapy or represented the natural course of the disease. Finally, although headache is not a classic symptom of the Miller Fisher syndrome, it has been described in a minority of patients.^13^

In summary, this case expands the phenotypic spectrum of the anti-GQ1b syndrome to include isolated cerebellar ataxia, suggesting that ataxia in this syndrome can have cerebellar origin. Clinicians should consider testing for anti-GQ1b antibodies in cases of acute cerebellar ataxia following infection.

## Data Availability

Data sharing is not applicable to this article as no datasets were generated or analysed during the current study.*

## Appendix

### List of Abbreviations

CSF: cerebrospinal fluid
EBV: Epstein-Barr virus
IVIG: intravenous immunoglobulin
MRI: magnetic resonance imaging.
